# Human parainfluenza virus type 1 regulates cholesterol biosynthesis and establishes quiescent infection in human airway cells

**DOI:** 10.1371/journal.ppat.1009908

**Published:** 2021-09-16

**Authors:** Yuki Kurebayashi, Shringkhala Bajimaya, Masahiro Watanabe, Nicholas Lim, Michael Lutz, Megan Dunagan, Toru Takimoto

**Affiliations:** Department of Microbiology and Immunology, University of Rochester Medical Center, Rochester, New York, United States of America; University of Wisconsin-Madison, UNITED STATES

## Abstract

Human parainfluenza virus type 1 (hPIV1) and 3 (hPIV3) cause seasonal epidemics, but little is known about their interaction with human airway cells. In this study, we determined cytopathology, replication, and progeny virion release from human airway cells during long-term infection *in vitro*. Both viruses readily established persistent infection without causing significant cytopathic effects. However, assembly and release of hPIV1 rapidly declined in sharp contrast to hPIV3 due to impaired viral ribonucleocapsid (vRNP) trafficking and virus assembly. Transcriptomic analysis revealed that both viruses induced similar levels of type I and III IFNs. However, hPIV1 induced specific ISGs stronger than hPIV3, such as MX2, which bound to hPIV1 vRNPs in infected cells. In addition, hPIV1 but not hPIV3 suppressed genes involved in lipid biogenesis and hPIV1 infection resulted in ubiquitination and degradation of 3-hydroxy-3-methylglutaryl-coenzyme A reductase, a rate limiting enzyme in cholesterol biosynthesis. Consequently, formation of cholesterol-rich lipid rafts was impaired in hPIV1 infected cells. These results indicate that hPIV1 is capable of regulating cholesterol biogenesis, which likely together with ISGs contributes to establishment of a quiescent infection.

## Author summary

Seasonal epidemics caused by parainfluenza viruses result in a significant burden of disease in children. These viruses infect airway epithelial cells and cause acute respiratory infection. Humans are the only known hosts for these viruses, but how these viruses are maintained within the population is not known. In this study, we analyzed human airway cells infected with type 1 and 3 parainfluenza viruses. Both viruses readily established persistent infection without causing major cytopathic effects. However, assembly and release of hPIV1 rapidly declined over time in sharp contrast to hPIV3. HPIV1 infected cells formed large aggregates of viral nucleocapsid at late time points, suggesting impaired nucleocapsid trafficking and virus assembly. Transcriptomic analysis of infected cells showed no major difference in IFN induction between the viruses, while hPIV1 induced elevated levels of interferon stimulated genes (ISGs) compared to hPIV3. Interestingly, hPIV1 infection specifically downregulated genes involved in cholesterol biogenesis. We also found that hPIV1 infection induced ubiquitination and degradation of 3-hydroxy-3-methylglutaryl-coenzyme A reductase, a rate limiting enzyme in cholesterol biosynthesis. These results suggest that induction of IFN-independent ISGs and suppression of cholesterol by hPIV1 likely play a role in establishing quiescent infection in human respiratory epithelial cells.

## Introduction

Parainfluenza viruses are one of the major causes of acute respiratory illnesses in infants, elderly and immunocompromised patients [1]. Among the four subtypes, type 3 parainfluenza virus (hPIV3) causes bronchiolitis, bronchitis, and pneumonia among young children, and type 1 (hPIV1) is the leading cause of croup in infants [2,3]. Although hPIV1 and 3 are similar in structure and genomic sequences, their epidemiological patterns are distinct. Population based studies over the past several decades show an occurrence of hPIV3 infections annually during spring while hPIV1 causes unique biennial outbreaks during fall in odd numbered years [1,3]. Humans are the only known host for both hPIV1 and 3, but how these viruses are maintained within the population and emerge during specific seasons is not known. Persistent infection can be a reason why these viruses are maintained during inter-epidemic periods. In fact, a few *in vitro* and *in vivo* analysis have shown that some paramyxoviruses can establish persistent infection [4,5]. Evidence showing an outbreak of parainfluenza virus infection at the South Pole after 10 and 29 weeks of complete social isolation also suggests persistent, asymptomatic infections [6,7]. Additionally, studies show viral persistent infection can cause chronic diseases, such as subacute sclerosing panencephalitis and postviral olfactory dysfunction in the case of measles virus and hPIV3, respectively [8,9]. Although persistent infection has a significant impact on viral epidemics and pathogenesis, few studies have been performed regarding understanding the mechanism by which hPIVs establish persistent infection.

Both hPIV1 and hPIV3 are non-segmented negative sense RNA viruses and belong to the genus *Respirovirus*. The viral genomes contain approximately 15,500 nucleotides and are organized to encode at least six common structural proteins (N, P/C, M, F, HN, and L). Viral genome replication and transcription take place entirely in the cytoplasm [2]. Progeny viral ribonucleocapsids (vRNPs) are formed in the cytoplasm and translocated to the site of assembly at the plasma membrane. All the structural components are translocated to plasma membrane lipid raft structures, where progeny virions are assembled and released by budding [10,11]. Translocation of vRNPs relies on host machinery involved in vesicular trafficking. We previously showed trafficking of hPIV1 and Sendai virus vRNPs by Rab11-regulated recycling endosomes [10,12,13]. We also showed that lipid rafts, and their essential component cholesterol, play a critical role in the assembly and stability of infectious progeny virions [14,15]. Treatment of human airway A549 cells with cholesterol reducing agents inhibited hPIV1 assembly and decreased virus particle formation and release, suggesting a critical role of cellular cholesterol in virus assembly [14]. Interestingly, some paramyxoviruses have been found to modulate cellular cholesterol biosynthesis during infection. A global transcriptional analysis of measles virus (MeV)-infected cells showed that most genes associated with the cholesterol biosynthesis pathway were down-regulated in persistently infected cells as compared to acutely infected cells [16]. It was also shown that 3-hydroxy-3-methylglutaryl-coenzyme A reductase (HMGCR), an important rate-limiting enzyme in the cholesterol biosynthesis pathway plays a critical role in several viral infections. HMGCR and low-density lipoprotein receptor, which are both involved in maintaining cellular cholesterol homeostasis, were found to be up-regulated during respiratory syncytial virus (RSV) infection [17]. Elevated HMGCR expression was correlated with RSV filament formation and F-actin mediated intracellular transmission of mature RSV particles, suggesting RSV may modulate cholesterol levels during virus infection for optimal growth [18].

In this study, we characterized type 1 and 3 hPIV infections in human airway cells *in vitro* for an extended period of time to determine viral replication and assembly, as well as cytotoxicity and found that these viruses can readily establish persistent infection. Further analysis on gene expression profiles of infected cells suggests differences in the ability of hPIV1 and 3 to regulate ISG expression. In addition, we found hPIV1 has a unique ability to suppress cholesterol biogenesis, which may contribute to reduced virus assembly and establishment of quiescent infection.

## Results

### hPIV1 and hPIV3 readily establish persistent infection in human airway cells

**Fig 1:**
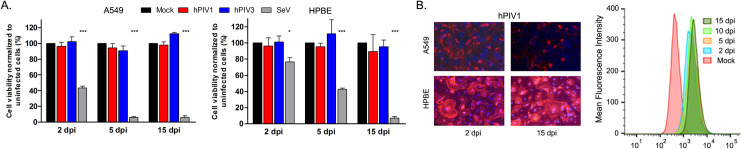
hPIV1 and hPIV3 persist in human airway cells without cytopathic effects. A549 or HPBE cells were mock-, hPIV1-, hPIV3-, or SeV-infected at an MOI of 5 and cultured for up to 15 days. (A) Cell viability was determined by cellular dehydrogenase assay (n = 3). *P≤0.05 ***P≤0.001. (B) Cell surface expression of viral HN was determined by immuno-fluorescence assay (left) or by flow cytometry analysis in A549 cells (right).

The majority of human respiratory viruses, such as influenza A virus and RSV, induce significant cytopathic effects on respiratory epithelial cells, but little studies have been done on the cytotoxicity of hPIVs in human airway cells. To determine if hPIVs induce lytic infection, A549 and human primary bronchial epithelial (HPBE) cells were infected with hPIV1, hPIV3 and Sendai virus (SeV, a murine parainfluenza virus type 1) at an MOI of 5 and cell viability was observed for 15 days. In contrast to SeV, which rapidly induced cell rounding and death starting at 2 days post infection (dpi), hPIV1 and hPIV3 did not affect cell viability even at 15 dpi (Fig 1A). All the cells infected with hPIV1 expressed HN protein at the cell surface even at 15 dpi suggesting that hPIV1 can readily establish persistent infection without any cytopathic effect (Fig 1B).

**Fig 2:**
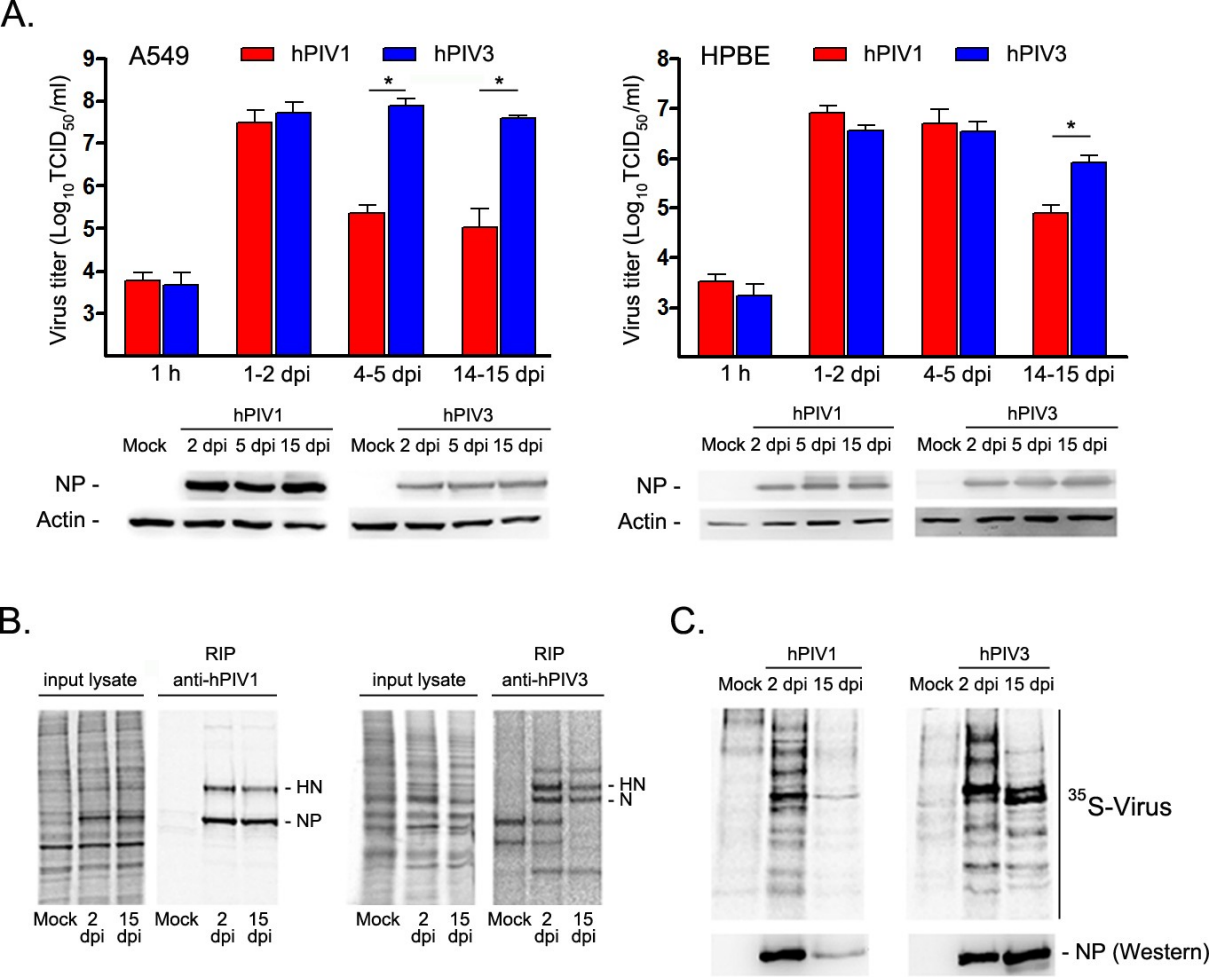
hPIV1, but not hPIV3 establishes quiescent infection in human airway cells. A549 or HPBE cells were mock-, hPIV1-, or hPIV3-infected at an MOI of 5 and cultured for up to 15 days. (A) Infectious progeny virions released into the medium for 24 h at various time points were titrated (n = 3). *P≤0.05. Accumulated NP proteins in cell lysates at each time point were determined by Western blotting. (B) *De novo* protein synthesis in A549 cells was determined by radio-immunoprecipitation after 3 h labeling with ^35^S-Met/Cys at 2 and 15 dpi. Radiolabeled viral HN and NP (or HN and N) were immunoprecipitated with specific mAbs or polyclonal serum. (C) Released and ^35^S-Met/Cys radio-labeled virus from A549 cells was purified by sucrose gradient centrifugation and analyzed by a Personal Molecular Imager after SDS-PAGE. The same samples were also tested for the presence of NP protein by Western blotting.

To determine if these infected cells shed infectious virions, supernatants from 24 h periods at 1–2, 4–5, and 14–15 dpi in A549 cells were collected and titrated for virus production. Interestingly, hPIV3 continuously produced high titers of infectious virus throughout the culture periods, while production of hPIV1 declined at late time points. Cells infected with hPIV1 produced about 100-fold less infectious virus between 4 to 5 or 14 to 15 dpi compared to that produced during 1–2 dpi (Fig 2A upper panel). A similar trend was observed in HPBE cells although suppression of hPIV1 release was observed at later time points compared to A549 cells. We next determined if this reduction is due to suppressed viral replication and protein production. Western blot analysis of viral NP in infected cells indicated no significant difference in accumulated NP at 2 and 15 dpi in hPIV1- or hPIV3-infected cells (Fig 2A lower panels). We further analyzed *de novo* viral protein synthesis by radioimmunoprecipitation (RIP) to determine if viral protein translation is reduced at later time points. Again, the results indicated no major difference in viral protein synthesis at 2 and 15 dpi for both viruses (Fig 2B). Next, we directly determined virus particle release from infected cells by metabolic labeling. We cultured infected cells with ^35^S-Met/Cys for 16 h and purified released virions from culture supernatants by ultracentrifugation through a sucrose cushion. Radiolabeled virion proteins were visualized by SDS-PAGE and phosphorimaging and also by Western blotting for NP (Fig 2C). The results clearly indicated limited formation and release of progeny hPIV1 virions at 15 dpi, suggesting that virion formation was blocked in hPIV1 infected cells at later time points even though similar levels of viral proteins were synthesized.

### Impaired hPIV1 assembly at late time points of infection

**Fig 3:**
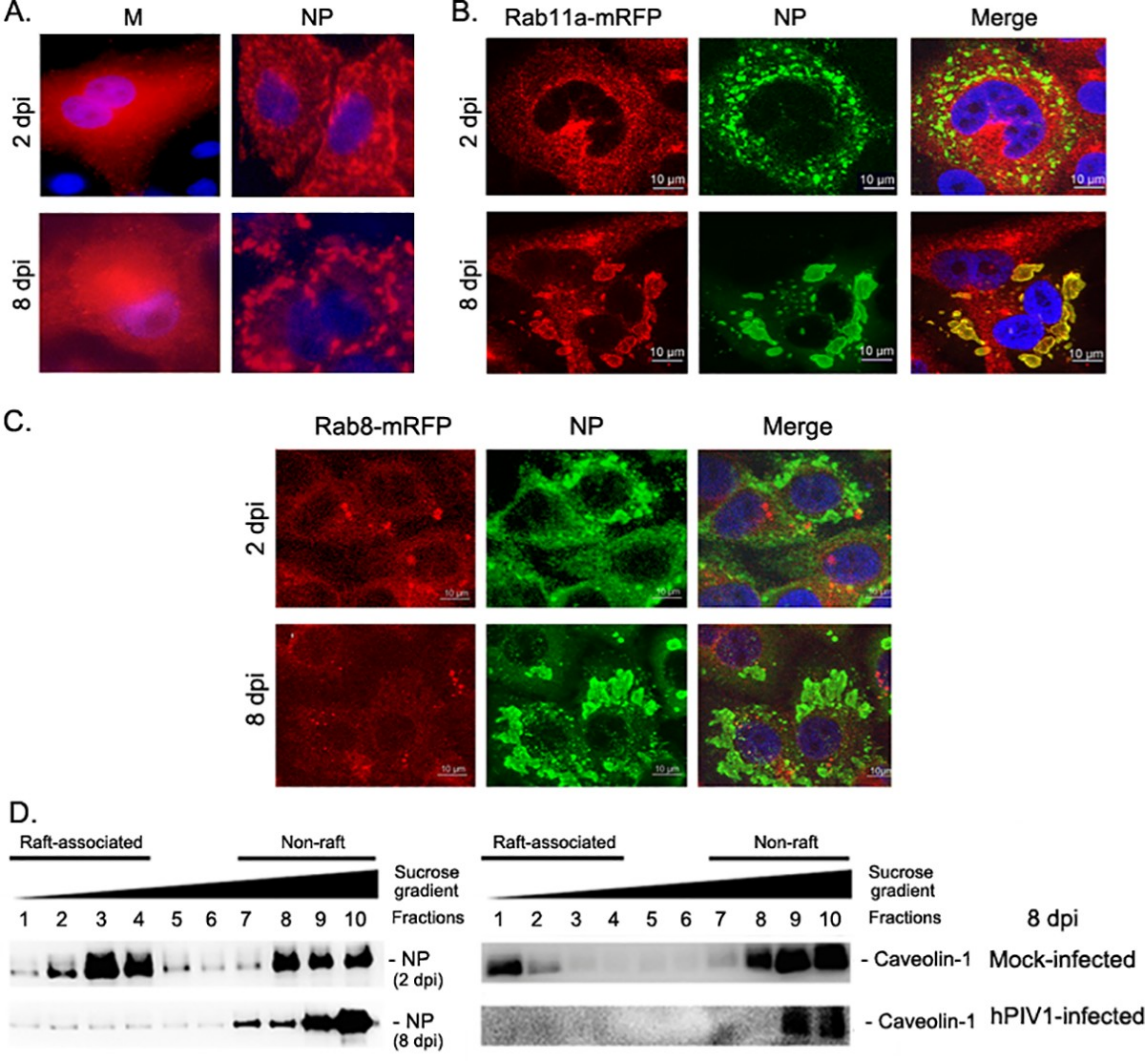
Impaired raft association and formation of large vRNP aggregates in hPIV1 infected cells at late time points. A549 cells were infected with hPIV1 at an MOI of 5 for 2 or 8 dpi. (A) IF analysis of hPIV1 M and NP at early and late time points. (B and C) A549 cells constitutively expressing Rab11- or Rab8-mRFP were infected with hPIV1 and analyzed at 2 and 8 dpi by IF. (D) Membrane floatation assay showing NP association with lipid raft at 2 and 8 dpi (left). Caveolin-1 association with lipid rafts was analyzed in mock and hPIV1 infected cells at 8 dpi (right).

To gain insights into how hPIV1 shedding was suppressed at later time points, we determined localization of viral structural proteins in hPIV1-infected A549 cells on days 2 and 8 post infection by immunofluorescence (IF) assay. Intracellular distribution of the viral matrix protein (M), which is involved in directing viral budding and release, did not change significantly during prolonged hPIV1 infection. However, the distribution of vRNPs identified by anti-NP Abs significantly changed from distinct punctae structure at 2 dpi to large aggregated forms detected at 8 dpi, suggesting impaired translocation of vRNPs to plasma membrane assembly sites and instead accumulation in the cytoplasm (Fig 3A). We previously showed that hPIV1 utilizes recycling endosomes to translocate vRNPs to apical surface assembly sites through specific interactions with Rab11a [12]. To determine if accumulated vRNPs in the cytoplasm is due to impaired vRNP interaction with Rab11a, we established A549 cells constitutively expressing mRFP-tagged Rab11a, as well as mRFP-tagged Rab8a as a control. Cells infected with hPIV1 were cultured for 2 and 8 days and colocalization of viral NP and Rab11a- or Rab8a-mRFP was determined by IF. At 8 dpi, the hPIV1 vRNPs aggregates co-localized with Rab11a-mRFP, suggesting that vRNPs still interacted with Rab11a even at late time points when viral shedding was significantly reduced (Fig 3B and 3C).

We next quantitated vRNP association with lipid raft domains at the plasma membrane of infected cells. Lysates of infected cells were collected at 2 or 8 dpi and membrane floatation assays were performed to determine the ratio of raft-associated and non-associated vRNPs. At 2 dpi, 54% of vRNPs were detected in raft-associated fractions (Fig 3D left panel). However, only 14% of vRNPs were detected in raft-associated fractions on 8 dpi, consistent with the observation of large vRNP aggregates in the cytoplasm at this time. To determine if raft formation was impaired during persistent hPIV1 infection, we quantitated raft association of cellular raft marker protein, caveolin-1. Raft association of caveolin-1 in hPIV1 infected cells on 8 dpi was significantly reduced compared to mock infected cells (Fig 3D right panel). These results suggest that hPIV1 infection reduced lipid raft formation at late time post infection.

### Differences in gene expression in hPIV1 and hPIV3 infected cells

To analyze how hPIV1 assembly and virion release were impaired in infected cells, we performed transcriptomic profiling of A549 cells uninfected or infected with hPIV1 or hPIV3 at an MOI of 3 and cultured for 2 or 10 days. Initial validation of the RNA samples from our primary dataset resulted in ~54–77 million sequencing reads per sample and mapped sequence reads for each sample ranged from 78–95% with uniquely mapped sequence reads ranging from 76–94% (S1A Fig). Sample-to sample distance matrix showed a high degree of similarity between the three replicates of each condition and some degree of similarity among all virus-infected conditions, but was highly distinct compared to mock samples (S1B Fig). Principal component analysis was performed to visualize the overall effect of experimental covariates and batch effects. The sample groups were separated following the PC1 and PC2 axes, indicating different gene expression profiles between mock and infected conditions and also between hPIV1 and hPIV3-infected conditions (S1C Fig).

**Fig 4:**
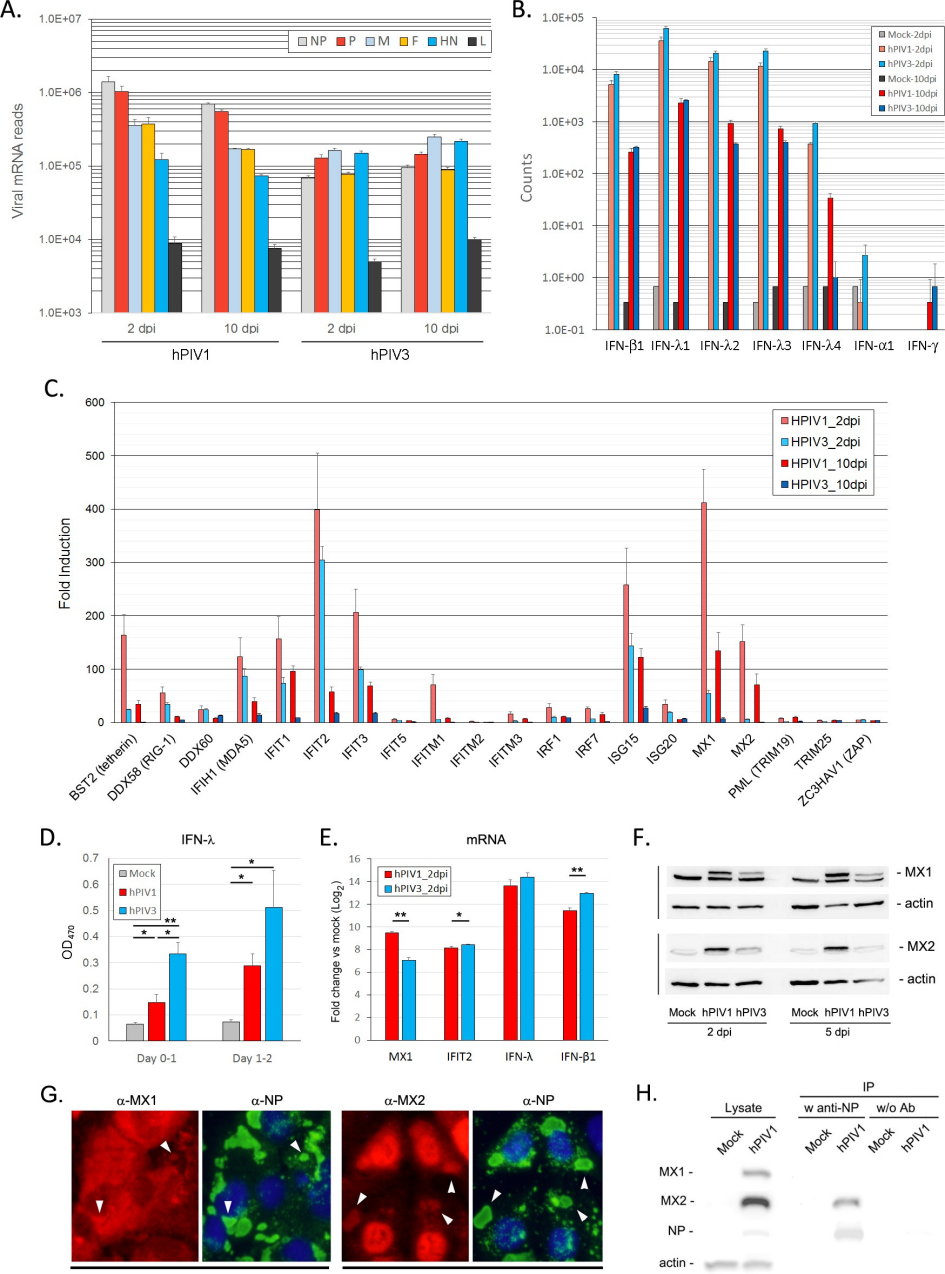
Expression of viral and host genes at 2 and 10 dpi. (A-C) A549 cells were mock-, hPIV1-, or hPIV3-infected at an MOI of 3 and total RNA was collected at 2 and 10 dpi for RNAseq analysis (n = 3). (A) Counts of viral mRNA reads. (B) Counts of IFN genes. (C) Fold induction of ISGs in hPIV1 or hPIV3 infected cells relative to mock. (D-H) A549 cells were mock-, hPIV1-, or hPIV3-infected at an MOI of 3. (D) Release of IFN-λ1 into culture medium was quantitated by ELISA (n = 3). *P≤0.05, **P≤0.01. (E) Expression of MX1, IFIT2, IFN-λ1 and IFN-β1 mRNAs in infected cells was determined by qRT-PCR (n = 3). Fold increase relative to mock after normalization to GAPDH is shown. *P≤0.05, **P≤0.01. (F) Expression of MX1, MX2 and β-actin in infected cell lysates was analyzed by immunoblotting. (G) Co-localization of MX proteins with vRNPs in hPIV1 infected cells at 5 dpi was determined by IF using anti MX1, MX2 or NP Abs. (H) Interaction of MX proteins with NP at 3 dpi was determined by immunoprecipitation of NP and immunoblotting to detect MX1, MX2 and viral NP.

Using our transcriptomic data, we first determined viral mRNA levels at 2 and 10 dpi. As expected from the protein expression in infected cells (Figs 1B and 2B), viral mRNA levels were not significantly changed, with only a 2 fold reduction at 10 dpi compared to 2 dpi in hPIV1 infected cells (Fig 4A). hPIV3 viral mRNAs were slightly increased at 10 dpi. These results indicate that unlike other paramyxoviruses such as measles virus or PIV5, hPIV1 and hPIV3 can maintain viral transcription even at 10 dpi [4]. Paramyxovirus mRNAs are transcribed by a stop-start process from their non-segmented linear genome, which typically leads to production of a transcriptional gradient where the most abundant mRNA is from the gene at beginning of the genome and the least abundant from the last. hPIV1 mRNAs showed a clear gradient as mRNA for the NP gene was most abundant followed by the rest of the genome. This gradient was less evident in hPIV3 transcripts although mRNA for the L gene was produced in the smallest quantity as expected.

Next, we determined differentially expressed host genes (DEGs) that were specifically upregulated or downregulated in hPIV1-infected cells compared to hPIV3-infected cells. DEGs between the hPIV1- and hPIV3-infected groups were analyzed under the threshold of p-value < 0.05. By comparing gene expression between hPIV1 and hPIV3 infection at 2 and 10 dpi, we detected 6,777 and 12,830 upregulated and 6,248 and 11,129 downregulated genes, respectively out of 172,085 with a non-zero total read count (S2 Fig).

DEGs upregulated in hPIV1 over hPIV3 infected cells were analyzed by gene ontology (GO) enrichment analysis using Enricher GO Biological Process 2018 database. Genes specifically upregulated in hPIV1-infected cells at 2 dpi were primarily related to cytokine mediated signaling pathways, but also included genes involved in cellular response to type I IFN and type I IFN signaling pathways (S1 Table). These pathways were also identified in the samples from 10 dpi (S2 Table), suggesting a difference in regulating innate immune signaling between hPIV1 and 3. To gain further insights into genes involved in specific inhibition of hPIV1 assembly, we focused on innate response genes, especially type I and III IFN and ISGs known to inhibit infection, replication or assembly of RNA viruses [19]. Both viruses strongly induced type I and III IFN expression at 2 dpi (Fig 4B). hPIV3 infection resulted in a stronger IFN induction compared to hPIV1. The level of IFN genes for both viruses declined at 10 dpi about 10 to 50-fold, although viral mRNA levels were not significantly different between 2 and 10 dpi (Fig 4A). We confirmed the production of similar amounts of IFNs at early times post infection by quantifying the released IFN-λ1 from supernatants of infected cells (Fig 4D).

In contrast to the IFN levels, significantly more ISGs were induced by hPIV1 compared to hPIV3 (Fig 4C). Some of these ISGs, such as BST2, MX1 and MX2, remained highly induced even at 10 dpi in hPIV1 infected cells. This pattern of innate immune response induction in infected cells was confirmed by qRT-PCR at 2 dpi (Fig 4E). Increased gene expression was also consistent with the protein levels of ISGs as determined by Western blot analysis (Fig 4F). MX1 and MX2 were expressed in hPIV1-infected cells much higher than hPIV3-infected cells. MX proteins have antiviral activity against various RNA viruses, but their effects on hPIV1 replication have not been demonstrated. Therefore, we analyzed if MX proteins interact with hPIV1 vRNPs in infected cells by IF. Both MX proteins co-localized with vRNP aggregates, especially MX2, which generally localizes to the nuclear membrane, but was detected in the cytoplasm and associated with vRNPs (Fig 4G). We further confirmed the interaction of MX2 with hPIV1 vRNPs by co-immunoprecipitation assay (Fig 4H). These results raise the possibility that MX2 affects the vRNP trafficking process and contributes to inhibition of hPIV1 assembly. In addition, we also determined if another ISG, cholesterol-25-hydroxylase (CH25H) inhibits hPIV1 assembly. CH25H is an enzyme that synthesizes the oxysterol 25-hydroxycholesterol (25HC) from cholesterol [20]. 25HC has been shown to have broad anti-viral activity by modifying plasma membrane compositions [21]. HPIV1 infected A549 cells were treated with 25HC at 5 or 12.5 μM immediately after infection and progeny virions were titrated at 48 hpi. No significant difference was found in virus titer between treated and untreated cells, suggesting that any modification of plasma membrane composition induced by 25HC at these concentrations is not sufficient to inhibit hPIV1 assembly and release (S3A Fig).

### hPIV1 infection downregulates cholesterol biogenesis

**Fig 5:**
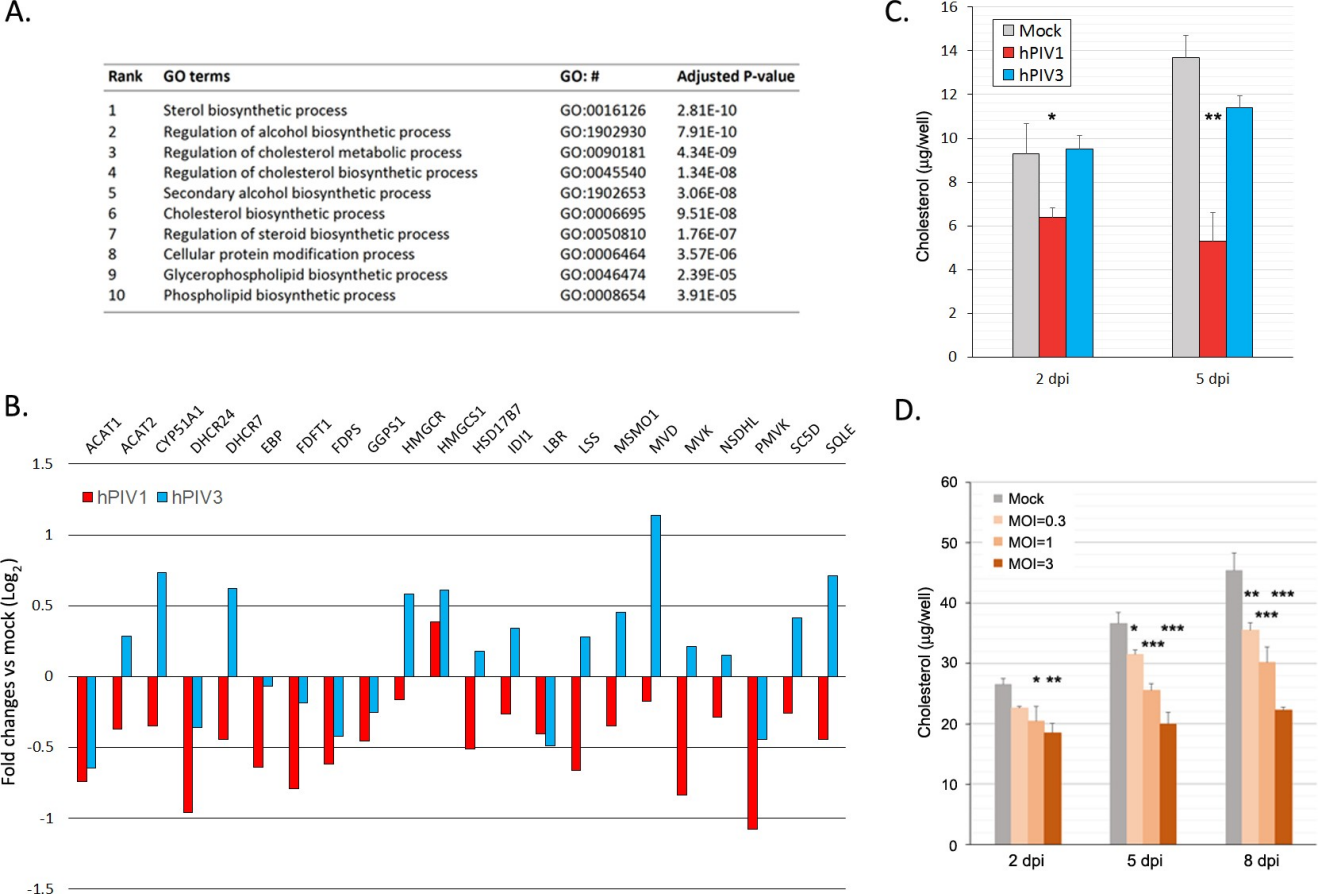
Downregulation of genes involved in cholesterol biogenesis in hPIV1 infected cells. (A) Top 10 GO terms of DEGs downregulated in hPIV1 infected cells compared to hPIV3. (B) Fold change in expression of genes involved in cholesterol biosynthesis in hPIV1 or hPIV3 infected cells compared to mock infected cells. (C) Total cellular cholesterol in A549 cells infected with hPIV1 or hPIV3 at an MOI of 5. (n = 3) *P≤0.05, **P≤0.01. (D) Total cellular cholesterol in A549 cells infected with various doses of hPIV1. (n = 3) *P≤0.05, **P≤0.01 ***P≤0.001.

We also performed GO enrichment analysis of genes specifically downregulated in hPIV1-infected cells at 2 dpi. Interestingly, genes involved in sterol and cholesterol biosynthetic and metabolic processes are the primary biological pathways specifically reduced by hPIV1 infection compared to cells infected with hPIV3 (Fig 5A and S3 Table). Most of the genes included in the superpathway of cholesterol biosynthesis (HumanCyc 2016, BioCyc ID: PWY66-5) were suppressed by hPIV1 infection (Fig 5B). The level of reduction of most of these genes was moderate but they are all commonly regulated by transcription factors known as sterol regulatory binding-element proteins (SREBPs), suggesting that hPIV1 may specifically manipulate SREBP activity [22]. However, this effect appears to be temporal because GO enrichment analysis of the 10 dpi samples did not show any significant difference in genes involved in sterol and cholesterol biosynthetic and metabolic processes (S4 Table).

Cholesterol is a key component of cellular lipid raft structures. Our previous study indicates that depletion of cellular cholesterol resulted in limited vRNP association with rafts and reduced virus assembly and release [14]. Therefore, we further analyzed the level of cellular cholesterol at various times after hPIV1 infection. Total cholesterol levels of cells infected with hPIV1 or hPIV3 were quantitated at 2 and 5 dpi. Compared to mock infected cells, hPIV1 infection resulted in 32 and 61% reduction at 2 and 5 dpi, respectively, while hPIV3 reduced total cholesterol by only 17% at 5 dpi (Fig 5C). This reduction of cellular cholesterol by hPIV1 was found to be dose dependent as determined by infections at MOI 0.3, 1 and 3, indicating that hPIV1 infection downregulates cellular cholesterol (Fig 5D).

**Fig 6:**
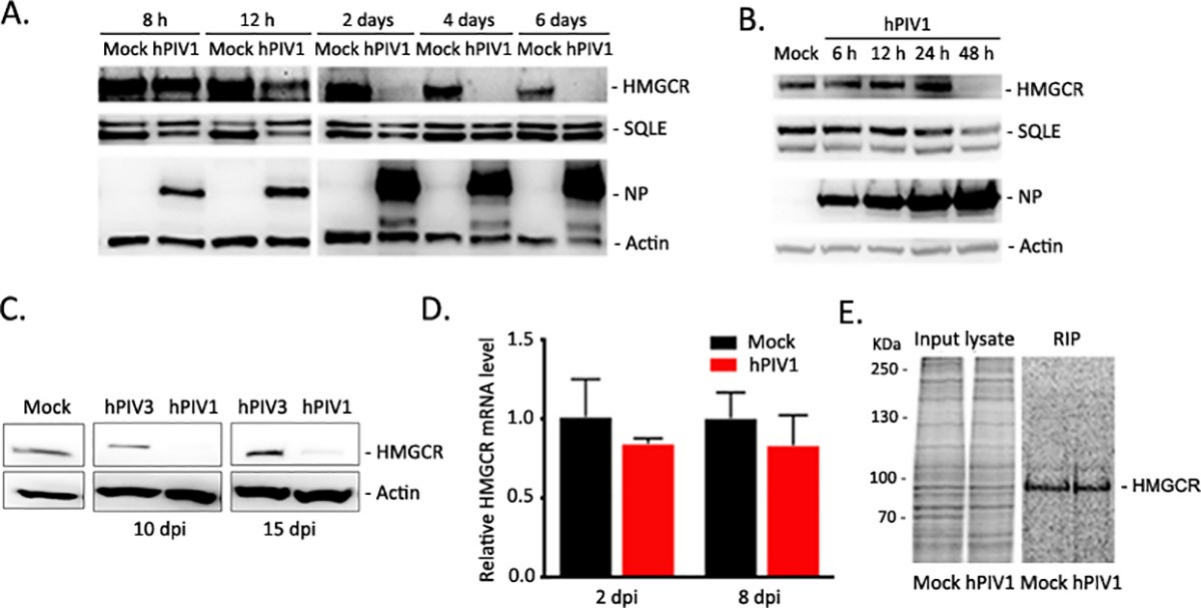
Reduced HMGCR expression in hPIV1-infected cells. (A) A549 cells were either mock- or hPIV1-infected at an MOI of 5 and expression levels of HMGCR, SQLE, viral NP and β-actin were assessed by immunoblotting. (B) HPBE cells were either mock- or hPIV1-infected at an MOI of 5 and expression levels of HMGCR, SQLE, viral NP and β-actin were assessed by immunoblotting. (C) A549 cells were either mock-, hPIV1-, or hPIV3-infected at an MOI of 5 and HMGCR and β-actin expression was assessed at late time points by immunoblotting. (D) A549 cells were infected with hPIV1 at an MOI of 5 and HMGCR transcripts were determined by qRT-PCR. HMGCR mRNA levels were normalized to GAPDH mRNA and relative mRNA levels are shown (n = 3). (E) A549 cells infected with hPIV1 at an MOI of 5 were labeled with ^35^S-Met/Cys for 3 h at 8 dpi and *de novo* HMGCR synthesis was analyzed by radio-immunoprecipitation.

To further analyze how hPIV1 maintains a low level of cholesterol in infected cells, we analyzed the expression of two key enzymes, HMGCR and squalene epoxidase (SQLE), which are involved in cholesterol biosynthesis. HMGCR is a rate limiting enzyme critical for cholesterol biosynthesis and a major target of cholesterol reducing drugs such as statins [23]. We used immunoblotting to determine the expression of HMGCR and SQLE in A549 cells infected with hPIV1 at various times after infection and compared to uninfected cells (Fig 6A). At 8 hpi, expression of HMGCR remained unchanged, however, a significant decrease in HMGCR expression in hPIV1 infected cells was observed starting at 12 hpi. Almost no HMGCR was detected starting at day 2 and up to 6 dpi, suggesting hPIV1 infection rapidly suppressed cholesterol biosynthesis within a few days after infection and maintained it at low levels at late time post infection. In contrast, SQLE levels in hPIV1 infected cells remained constant throughout the same time. Similar downregulation of HMGCR was observed in HPBE cells, although the loss of HMGCR expression was slower than that observed in A549 cells (Fig 6B). This loss of HMGCR was detected even at 10 and 15 dpi in hPIV1 but not hPIV3 infected A549 cells (Fig 6C), which likely contributes to the low level of cellular cholesterol found in hPIV1 infected cells.

Almost complete loss of HMGCR protein levels in hPIV1 infected cells cannot be explained due to suppressed HMGCR gene expression because our transcriptomic analysis indicates only a slight reduction of HMGCR mRNA (Fig 5B). Independent quantification by qRT-PCR analysis confirmed only a slight reduction of HMGCR mRNA expression at 2 and 8 dpi in A549 cells (Fig 6D). Therefore, we determined if *de novo* HMGCR protein synthesis was inhibited in hPIV1 infected cells at a late time point. A549 cells infected with hPIV1 and cultured for 8 days were labeled with ^35^S-Met/Cys for 3 h and newly synthesized HMGCR was analyzed by RIP using an HMGCR specific antibody. We detected no major difference in HMGCR synthesis between infected and uninfected cells at 8 dpi (Fig 6E). These results suggest that hPIV1 infection does not significantly affect HMGCR mRNA expression or protein synthesis.

### Ubiquitination and degradation of HMGCR in hPIV1-infected cells

**Fig 7:**
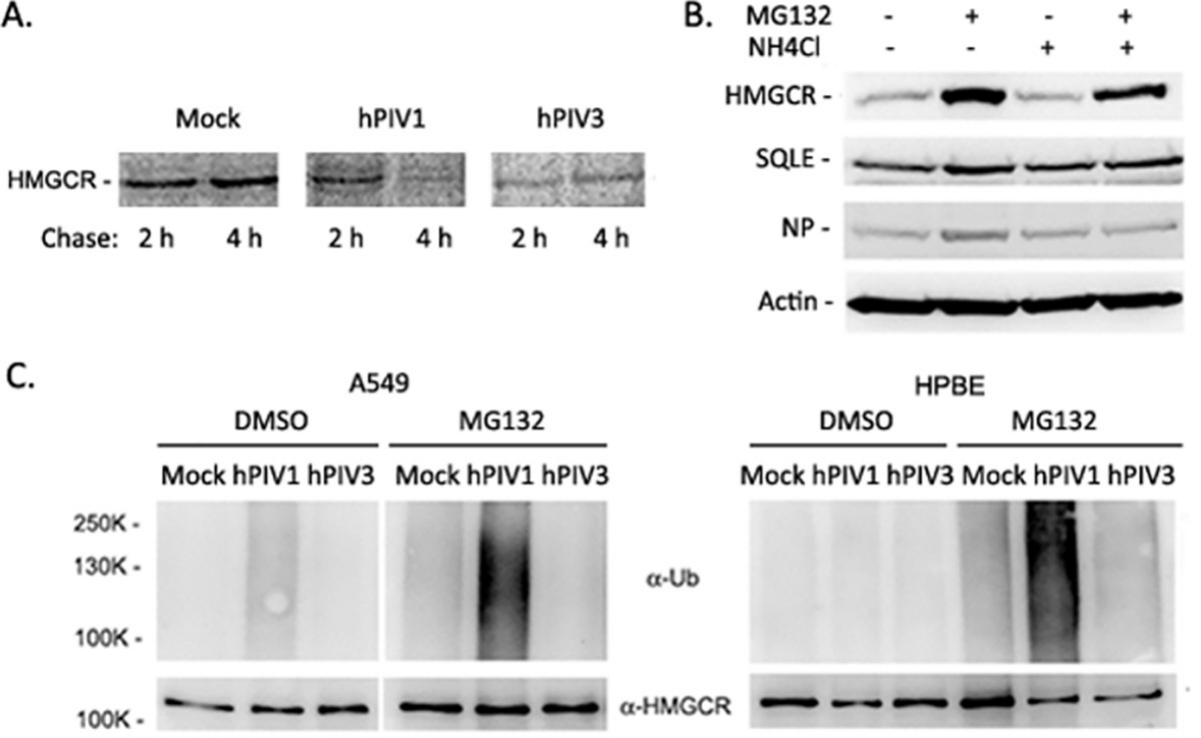
Ubiquitination and proteasomal degradation of HMGCR in hPIV1 infected cells. (A) A549 cells were infected with hPIV1 or hPIV3 at an MOI of 5 for 2 days. Cells were then labeled with ^35^S-Met/Cys for 30 min and chased for either 2 or 4 h. HMGCR stability was analyzed by radio-immunoprecipitation. (B) A549 cells infected with hPIV1 at an MOI of 5 were treated with either MG132 (10 μM) and/or NH4Cl (20 mM) at 8 hpi for 8 h. Expression of HMGCR, SQLE, viral NP and β-actin were analyzed by immunoblotting. (C) A549 or HPBE cells were mock- or hPIV1-, hPIV3- infected at an MOI of 3 for 18 h. MG132 (10 μM) or DMSO was added and cells were cultured for an additional 8 h. HMGCR in cell lysates was immunoprecipitated using an HMGCR specific Ab and immunoblotting was performed to detect ubiquitin or HMGCR.

The process of cholesterol metabolism is regulated at multiple levels including transcription, translation, and enzymatic activity [24]. Previous studies also showed that ubiquitination and degradation of HMGCR is a major negative feedback regulatory mechanism governing cholesterol biosynthesis [25–27]. Excess cholesterol can trigger degradation of HMGCR through the ubiquitin-proteasome pathway and proteasome inhibition blocks this process, leading to an accumulation of ubiquitinated HMGCR [27,28]. We first determined whether reduced HMGCR expression was due to protein degradation. HMGCR stability was determined by a pulse-chase experiment in A549 cells, which were labeled for 30 min and chased for 2 or 4 h. The results showed that HMGCR was rapidly degraded in hPIV1-infected cells but not in mock or hPIV3-infected cells (Fig 7A). We next tested if hPIV1 infection induces the ubiquitin-proteasome pathway to degrade HMGCR, which can be blocked by the proteosomal degradation inhibitor MG132. A549 cells infected with hPIV1 for 16 h were treated with MG132 or the lysosomal degradation inhibitor NH_4_Cl for an additional 8 h, and then HMGCR protein levels were analyzed by immunoblotting. Treatment with MG132 but not NH_4_Cl led to the accumulation of HMGCR in hPIV1-infected cells, suggesting hPIV1 infection induces proteosomal degradation of HMGCR (Fig 7B). Next, we determined if HMGCR was ubiquitinated in hPIV1 infected cells. Mock or virus infected cells were treated with MG132 and HMGCR was immunoprecipitated using a specific antibody and applied for western blot analysis with anti-ubiquitin Ab. Ubiquitination of HMGCR was detected in hPIV1, but not mock or hPIV3-infected cells (Fig 7C left). The same results were obtained in infected HPBE cells (Fig 7C right), showing that hPIV1 infection induces ubiquitination and subsequent degradation of HMGCR. Taken together, these data suggest that hPIV1 triggers ubiquitination and proteasomal degradation of HMGCR to reduce cellular cholesterol. To determine if inhibition of HMGCR degradation by MG132 is sufficient to rescue hPIV1 assembly and virion production, we treated hPIV1 infected A549 cells immediately after infection and cultured for 5 days in the presence of MG132. We then titrated progeny virions released into the supernatant over the 24 hour period from 4–5 dpi. Treatment with MG132 was not sufficient to rescue hPIV1 viral titers (S3B Fig). Overall, our data indicate that hPIV1 infection influences multiple signaling pathways, including ISG induction and cholesterol biogenesis, to inhibit virus assembly and allow for the establishment of a quiescent infection in human airway epithelial cells.

## Discussion

Persistent RNA virus infections have been shown to be associated with chronic diseases [29]. Viruses causing acute respiratory infections are suggested to induce persistent, quiescent or latent infections. Some paramyxoviruses, such as RSV, hPIV3 and parainfluenza virus type 5 (PIV5), have been shown to readily establish persistent infections in cultured cells [4,30–33]. To establish persistent infection, the virus needs to avoid killing infected cells while maintaining viral genomes in the form of nucleocapsids. In this study, we have analyzed interactions between hPIV and human airway cells and dissected the molecular mechanisms by which hPIV1 can establish long-term infections in human airway cells. Unlike closely related SeV, hPIV1 and hPIV3 did not induce significant cytopathic effects even at 15 dpi when infected cells were cultured in serum containing medium (Fig 1). In this culture condition, progeny virions are not infectious due to the absence of the enzyme that activates the viral F protein, meaning no additional rounds of infection occur after the initial infection. Under these conditions, hPIV1 and hPIV3 can maintain their genomes in human airway cells without killing or triggering host responses that lead to cell death. Remarkably, the viruses continue to produce transcripts and viral proteins within the quiescently infected cells (Figs 2B and 4A). This ability to maintain viral genomes within quiescently infected cells may explain how these viruses are maintained within the population and cause seasonal outbreaks [5].

Despite continuous viral protein synthesis, infectious hPIV1 production gradually decreased as opposed to hPIV3 (Fig 2). We further investigated hPIV1 infected cells to determine how progeny virion assembly and release were specifically down regulated compared to hPIV3 infected cells. In hPIV1 infected cells, we observed 1) formation of large vRNP aggregates associated with Rab11a and reduced vRNP lipid raft association (Fig 3), 2) induction of some unique ISGs (Fig 4C), and 3) downregulation of genes involved in cholesterol biogenesis (Fig 5B) alongside ubiquitination and degradation of the key cholesterol biosynthesis enzyme HMGCR (Figs 6 and 7). Newly synthesized hPIV1 vRNPs are transported to the plasma membrane via Rab11a-mediated recycling endosomes [12]. Impaired vRNP trafficking could be due to the loss of vRNP interaction with Rab11a, but our data indicate that Rab11a still associates with vRNPs (Fig 3B). These results suggest that the interaction between Rab11 and vRNP remains unchanged during prolonged infection and decreased virus production may not be due to alteration of Rab11 mediated recycling endosomes. The fact that hPIV1 infection induced higher levels of some unique ISGs compared to hPIV3 may implicate some of these ISGs in specifically inhibiting trafficking of progeny vRNPs to the assembly site. These ISGs, which include MX1, MX2 and BST2, were found to be upregulated in hPIV1-infected cells by at least 7-fold compared to hPIV3 infected cells at both early and late time points after infection (Fig 4C). It is not known if these ISGs block the assembly process of hPIV1. However, we detected co-localization of MX proteins, and specifically binding of MX2, to vRNPs (Fig 4G and 4H). This raises the possibility that MX2 induced in hPIV1 infected cells contributes to the sequestration of vRNPs and inhibition of hPIV1 assembly.

Importantly, we found that hPIV1 specifically suppresses the biosynthesis of cholesterol through downregulating genes involved in the synthesis pathway and by inducing proteosomal degradation of the key enzyme HMGCR. Cholesterol is a major component of lipid raft structures in the plasma membrane, which are the assembly sites for many enveloped viruses [10,34]. Depletion of cellular cholesterol reduces vRNP association with lipid raft membranes and production of hPIV1 [14]. This reduction of cholesterol biogenesis in hPIV1 infected cells may explain reduced lipid raft membrane formation and impaired trafficking of vRNPs (Fig 3). Expression of most cholesterol biogenesis genes are controlled by SREBP transcription factors [35]. SREBPs are synthesized as inactive precursors and are associated with SREBP cleavage-activating protein (SCAP) and Insulin-induced gene protein (INSIG) in the ER. Upon appropriate conditions, such as low sterol concentration, the interaction of INSIG and SCAP decreases to allow the translocation of the SREBP-SCAP complex to the Golgi where the N-terminal domain of SREBP is cleaved and translocated to the nucleus to regulate expression of target genes [36]. Most of the cholesterol biogenesis genes regulated specifically by SREBP2 are suppressed in hPIV1 infected cells (Fig 5B), thus it is possible that hPIV1 regulates SREBP2 activity. Additionally, hPIV1 infection induced degradation of HMGCR (Fig 6). HMGCR catalyzes the conversion of HMG-CoA to mevalonate, a critical step that leads to synthesis of cholesterol and other nonsterol isoprenoids. Accumulation of sterols in ER membranes leads to the accelerated ubiquitination and proteasomal degradation of HMGCR. INSIG proteins are also required for the sterol-induced degradation of HMGCR. INSIG1 is known to associate with ubiquitin ligases including GP78, TRC8 and RNF145 [27,37,38]. Under sterol repletion conditions, INSIG1 binds to the membrane domain of HMGCR and triggers HMGCR ubiquitination, potentially through these previously mentioned ubiquitin ligases [36]. We found that degradation of HMGCR in hPIV1 infected cells continued to at least 15 dpi (Fig 6C). Since INSIG proteins are involved in both SREBP activation and HMGCR degradation, it is possible that hPIV1 directly or indirectly regulates the activity of INSIG proteins to suppress cholesterol biosynthesis. This ability to regulate cholesterol biogenesis may not be unique to hPIV1. A previous study on cells persistently infected with measles virus also found the downregulation of cholesterol synthesis genes, suggesting a possible link with defective viral assembly and budding during persistent infection [16]. Based on these results, regulation of cholesterol biosynthesis could be a major mechanism of how hPIV1 is maintained in the human population. Consistent with this, some clinical studies report that statin use is associated with reduced mortality in patients hospitalized with respiratory viral infections [39–41]. However, other host antiviral responses we detected in hPIV1 infected cells may also contribute to the decreases assembly and release of progeny virions. Considering the fact that humans are the only known host for hPIV1 and that outbreaks occur seasonally, virus induced cellular responses and regulation of sterol biogenesis likely play a key role in establishing quiescent infection.

## Materials and methods

### Cells and viruses

LLC-MK2 (ATCC, CCL-7) and A549 cells (ATCC, CCL-185) were maintained in Dulbecco’s modified Eagle’s medium (DMEM; Corning) supplemented with 8% fetal calf serum (FCS; Life Technologies), 25mM HEPES (Gibco) and 50 μg/ml Gentamicin (Life Technologies). Primary normal human bronchial/tracheal epithelial (HPBE) cells (ATCC, PCS-300-010) were maintained in airway epithelial cell basal media (ATCC, PCS-300-030) supplemented with bronchial/tracheal epithelial cell growth kit components (ATCC, PCS- 300–040). A549 cells constitutively expressing Rab11-mRFP or Rab8-mRFP were established as described before for HeLa cells [13]. Briefly, A549 cells were transfected with the mRFP-Rab11a or mRFP-Rab8a cDNA in pCAGGS-HygR vector and hygromycin resistant cells were selected and grown in DMEM containing 8% FCS (DMEM-FCS). SeV (strain Enders), hPIV1 (strain C-35) and hPIV3 (strain C243, ATCC VR-93) were grown in LLC-MK2 cells in DMEM supplemented with 0.15% bovine serum albumin (DMEM-BSA) and acetylated trypsin at 2 μg/ml.

### Cell viability and cholesterol assays

A549 or HPBE cells infected with the viruses were cultured in DMEM-8% FCS at 34°C. Culture medium was replaced with fresh medium every 2 days. Cell viability was measured using cellular dehydrogenase based assay, Cell Counting Kit-8 (Dojindo) according to the manufacturer’s protocol. Total quantity of cellular cholesterol of infected cells in 12-well plates were measured by Amplex Red Cholesterol assay (Invitrogen).

### Immunofluorescence (IF) assays

Infected cells were fixed with 4% paraformaldehyde (PFA) in PBS for 30 min at room temperature (RT) and reacted with anti-HN monoclonal antibody (mAb) cocktail (P18, P24 and P37) [42] and anti-mouse IgG-Alexa Fluor 594 (Thermo Fisher). Localization of hPIV1 M and NP proteins were analyzed using anti-hPIV1 M or NP (P19, P27, P35) mAbs after permeabilizing the cells with 0.2% Triton X-100 in PBS for 10 min. Co-localization of Rab11a or Rab8a with viral proteins were performed using A549 cells constitutively expressing Rab11a-mRFP or Rab8a-mRFP. Co-localization between NP and MX1 or MX2 was determined using rabbit anti-MX1 and anti-MX2 polyclonal antibodies (Proteintech and Novus, respectively). Fixed cells were reacted with mouse anti-NP mAb cocktail together with rabbit anti-MX1 or MX2 Ab followed by anti-mouse IgG-Alexa Fluor 488 and anti-rabbit IgG-Alexa 594. Fluorescent images were acquired using an Olympus FV1000 confocal microscope or an Olympus IX 50 inverted fluorescence microscope.

### Flow cytometry analysis

A549 cells uninfected or infected with hPIV1 at 2, 5, 10 and 15 dpi were trypsinized and fixed using 4% PFA for 30 min at RT. After brief centrifugation, non-permeabilized cells were stained with anti-hPIV1 HN (P18, P24 and P37) antibodies for 1 h. Donkey anti-mouse IgG conjugated with Alexa Fluor 488 (Invitrogen) was used as a secondary antibody. Fluorescent signals was recorded with a 525/50 band-pass filter and excitation using a 488-nm laser. Color LSR-II Flow Cytometer (BD Biosciences) were used for all flow cytometry analysis. Flow cytometry data were processed using FlowJo software.

### Viral replication and progeny virus production

To titrate infectious virus production, cells were infected with hPIV1 at an MOI of 5 for 1 h and cultured in DMEM-FCS for up to 15 days. Culture media was replaced with fresh medium every day. Twenty-four h before harvest, the medium was replaced with DMEM-BSA. Culture supernatants containing progeny virions produced during the 24 h periods were collected at the indicated time points and virus titers were measured after treatment with TPCK-treated trypsin at 5 μg/ml for 1 h. To analyze the effect of 25HC on virus production, infected cells were treated with 25HC (5 μM or 12.5 μM) or DMSO for 24 h in DMEM-FCS. Culture medium was replaced with DMEM-BSA containing the same amount of 25HC or DMSO and incubated for 24 h. Virus titer in the supernatant was then determined by IF using anti-NP mAb. To analyze the effect of MG132 on virus production, infected cells were cultured with MG132 (3 μM or 10 μM) or DMSO in DMEM-FCS for 4 days. The medium was then replaced with DMEM-BSA containing same amount of MG132 or DMSO and cultured for 24 h. Virus titer in the supernatant was measured as above. For the analysis of radiolabeled virus, infected cells in 6-well plates were cultured in labeling medium containing 50 μCi of ^35^S-Met/Cys (PerkinElmer) for 16 h. Labeled cell lysates were used for immunoprecipitation with a cocktail of mAbs against hPIV1 HN and NP or anti-hPIV3 guinea pig serum (BEI, NR-3235) coupled to Protein G Dynabeads (Thermo Fisher). Released virions in supernatants (1.5 ml) were purified by step gradient centrifugation using 5% and 40% sucrose in TNE buffer (25 mM Tris HCl, 150 mM NaCl and 4 mM EDTA, pH 7.5) at 210,000 x *g* for 2 h in a SW44Ti rotor (Beckman). One ml of virions was collected from the interface and re-suspended in 3 ml of PBS followed by ultracentrifugation at 210,000 x *g* for 2 h at 4°C in a SW44Ti rotor. The virus pellet was re-suspended in 2x NuPAGE LDS sample buffer (Thermo Fisher) and resolved by SDS-PAGE. Fixed and dried gels were exposed onto a phosphor screen and visualized using a Personal Molecular Imager and Quantity One software (Bio-Rad).

### Western blot analysis

To determine viral protein synthesis, infected cells were lysed with TNE buffer and resolved by SDS-PAGE. After transferring to PVDF membranes, blots were reacted with anti-hPIV1 NP mAbs cocktail (P19, P27, P35) or anti-hPIV3 guinea pig serum. For the analysis of HMGCR or SQLE expression, anti-HMGCR (Atlas) or anti-SQLE (Santa Cruz) antibodies were used. For the detection of MX1 and MX2, rabbit anti-MX1 and anti-MX2 polyclonal antibodies were used. Anti-β-actin mAb (Cell Signaling) was used for a loading control. Band intensities were quantitated using BioRad Quantity One software.

### Detection of *de novo* viral protein synthesis

Uninfected or infected cells were radiolabeled using ^35^S-Met/Cys (PerkinElmer) for 3 h at 2 and 15 dpi. De novo viral protein synthesis was analyzed by RIP using a cocktail of anti-hPIV1 NP (P19, P27, P35) and HN (P18, P24 and P37) mAbs or guinea pig anti-hPIV3 serum and resolved by SDS-PAGE. Fixed and dried gels were exposed to a phosphor screen and visualized using a Personal Molecular Imager and Quantity One software (Bio-Rad).

### Raft flotation assays

Raft flotation assays were performed as described previously with a few modifications [43]. Cells were harvested by scraping and pelleted by low speed centrifugation (2,000 x *g* for 3 min). Following re-suspension in 300 μl of cold hypotonic TE buffer (10 mM Tris HCl, pH 7.5, 4mM EDTA), cells were passaged 30 times through a 27 ½ gauge hypodermic needle and centrifuged at 1,500 x *g* for 3 min. Next, 100 μl of cold TNE buffer containing 1% Triton-X 100 was added to 300 μl of lysate and incubated on ice for 40 min. Each lysate (400 μl) was mixed with 700 μl of 70% sucrose in TNE with 1% Triton-X 100 and overlaid on layers of 2 ml 30% sucrose and 1 ml 2.5% sucrose in 1x TNE containing 1% Triton-X 100. After centrifugation at 26,000 x *g* for 18 h at 4°C in an SW55-Ti rotor, 400 μl fractions were collected from the top. Proteins in the samples were precipitated with Trichloroacetic acid (TCA), re-suspended in 2x NuPAGE LDS sample buffer and resolved by SDS-PAGE, followed by immunoblot analysis using anti-hPIV1 NP or anti-caveolin-1 mAbs (Novus). Band intensities were quantitated using BioRad Quantity One software.

### RNA-Seq analysis

Confluent A549 cells in a 12-well plate were either left uninfected or infected with hPIV1 or hPIV3 at an MOI of 3 for 2 or 10 days in triplicates. Total RNAs were extracted using Illustra RNAspin Mini (GE Healthcare). RNA concentration and quality were measured using an Agilent 2100 Bioanalyzer, showing RNA Integrity Number (RIN) values of > 7.0, OD260/280 = 2.0–2.2 and 28S: 18S rRNA > 1.0. mRNA was purified from 200 ng total RNA with oligo-dT magnetic beads and fragmented. First-strand cDNA synthesis was performed with random hexamer priming followed by second-strand cDNA synthesis. End repair and 3’ adenylation was then performed on the double-stranded cDNA. Illumina adaptors were ligated to both ends of the cDNA, purified by gel electrophoresis, and amplified with PCR primers specific for the adaptor sequences to generate amplicons of approximately 200–500 bp in size. The amplified libraries were hybridized to the Illumina single-end flow cell and amplified using an Illumina cBot at a concentration of 8 pM per lane. Single-end reads of 100 nt were generated for each sample and aligned to the organism-specific reference genome. RNA-sequencing was performed using an Illumina HiSeq2500 sequencer.

Sequence reads were cleaned and adapter trimmed using trimmomatic-0.36. The reads were quality checked by FastQC before mapping them to the human reference genome (hg38_gencode31), hPIV1 (Accession: NC_003461.1) or hPIV3 genomes (NC_001796.2) with STAR_2.7.0f or Salmon (ver.0.13.1). Raw read counts were obtained using featurecounts from the subread-1.6.4 package and gencode 25 human gene annotations using only uniquely aligned reads. DESeq2-1.22.1 within R-3.5.1 was used to perform data normalization and differential expression analysis with an adjusted p-value threshold of 0.05 on each set of raw expression measures. The ‘lfcShrink’ method was applied, which moderates log2 fold-changes for lowly expressed genes.

Functional enrichment analysis of DEGs was performed with Enrichr analysis tool (http://amp.pharm.mssm.edu/Enrichr/). To associate a set of DEGs with a functional biological term, Gene Ontology terms were assigned using GO biological processes 2018 annotation set. GO terms were ranked by a combined score (ln(P-value) × Z-score); the p-value was computed using the Fisher exact test.

### Quantitation of mRNAs by qRT-PCR

Cells uninfected or infected with the viruses at an MOI of 3 were collected and total RNA was extracted using illustra RNAspin Mini kit (Cytiva) or Trizol (Invitrogen) following the manufacturer`s protocol. cDNAs were synthesized using oligo(dT)20 primer with the SuperScript III first-strand synthesis system (Life Technologies). Real-time PCR (qPCR) was performed using primers specific for target genes, and 2x Power SYBR green PCR master mix (Applied Biosystems). PCR was performed using a 7300 real-time PCR system (Applied Biosystems) with the following cycle conditions; 95°C for 10 min, followed by 40 cycles of 95°C for 15 s and 60°C for 1 min. Primers that were designed to amplify specific mRNAs were as follows. HMGCR: GTCATTCCAGCCAAGGTTGT (forward) and CATGGCAGAGCCCACTAAAT (reverse). SQLE: AAACTTGGTGGCGAATGTGT (forward) and ACACGGCATAGATTGCAACA (reverse). MX1: ACCTACAGCTGGCTCCCGAA (forward) and GCACTCAAGTCGTCAGTCCA (reverse). IFIT2: TGCCTCCTTCCTCCTCCT (forward) and CCTGTTCTCTGCCCTCGT (reverse). IFN-λ: CTGGAGGCATCTATCACCTT (forward) and AGTAGGGCTCAGCGCATAAA (reverse). IFN-β1: TTACAGGTTACCTCCGAAACTGAA (forward) and GGTTGAAGAATGCTTGAAGCAA (reverse). GAPDH: ACCCAGAAGACTGTGGATGG (forward) and TTCTAGACGGCAGGTCAGCT (reverse). Copy numbers of the housekeeping gene GAPDH were measured in each sample for normalization.

### Quantification of IFN-λ1

A549 cells were mock infected or infected with hPIV1 or hPIV3 at an MOI of 3 in 12-well plates and cultured at 34°C. Culture supernatant was collected every 24 h at 24h and 48h post-infection. Released IFN-λ1 was measured using IL-29 Human Uncoated ELISA Kit (Thermo) according to the manufacturer’s instructions.

### Pulse-chase analysis and ubiquitination of HMGCR

A549 cells were infected with hPIV1 or hPIV3 at an MOI of 5 and cultured for 2 days. Washed cells were labeled with ^35^S-Met/Cys for 30 min and chased for either 2 or 4 h with DMEM-8% FCS. HMGCR in the cell lysates was immunoprecipitated using anti-HMGCR Ab coupled to Protein G Dynabeads. To analyze HMGCR degradation post infection, cells were infected at MOI 5 for 8 h and treated with proteosomal degradation inhibitor MG132 (10 μM) or lysosomal degradation inhibitor NH_4_Cl (20 mM) for an additional 8 h and HMGCR protein levels were analyzed by immunoblotting. For the analysis of HMGCR ubiquitination, cells infected with hPIV1 or hPIV3 at an MOI of 3 were cultured for 18 h at 34°C. MG132 (10 uM) or DMSO was added and cells were cultured for an additional 8 h. HMGCR in the cell lysates was immnoprecipitated as described above and analyzed for ubiquitination by Western blotting using mouse anti-ubiquitin antibody (Santa Cruz).

### Co-immunoprecipitation assay

A549 cells infected with hPIV1 were cultured for 3 days and lysed with Pierce IP lysis buffer (ThermoFisher). vRNP in the lysates were immunoprecipitated using anti-NP mAb coupled to Protein G Dynabeads. Washed materials were applied for Western blot analysis and MX1, MX2 and NP were detected as above.

### Statistical analysis

A minimum of 3 biological replicates was preformed for each experiment. Statistical analysis was performed in GraphPad Prism 9.0 using either one-way analysis of variance, followed by Tukey’s post-hoc test or a two-tailed t-test assuming equal variance. All quantitative data is represented as the mean ± standard deviation of the indicated number of experiments. Significant differences between groups of data are represented as *P≤0.05, **P≤0.01, or ***P≤0.001.

## Supporting information

S1 Fig Processing statistics of transcriptomic data (RNA sequencing) and validation of RNA samples. (A) Processing statistics for transcriptomic analysis of the 18 RNA samples including three biological replicates of uninfected (mock), hPIV1- or hPIV3-infected A549 cells cultured for 2 or 10 days. (B) Sample-to-sample distance matrix of RNA-Seq analysis. A heatmap shows the hierarchically clustered Euclidean distances between samples from the regularized log transformation of the normalized count data. The scale on the right demonstrates the arbitrary unit of distance between samples in which the dark color represents less distance (more similarity) and the light color represents greater distance between samples (less similarity). (C) Principal component analysis (PCA) plot representing the variance in the gene dataset. The samples shown in the 2D plane are spanned by their first two principal components of all samples including three replicates of mock, hPIV1-or hPIV3-infected conditions.
(TIF)

S2 Fig Number of DEGs that were specifically upregulated or downregulated under the threshold of p-value < 0.05. (TIF)

S3 Fig Effect of 25HC and MG132 on hPIV1 production. A549 cells were infected with hPIV1 at an MOI of 3 for 1 h and cultured in the presence of 25HC (A) or MG132 at indicated concentrations. Quantities of infectious virions in supernatants produced during 1–2 dpi (A) or 4–5 dpi (B) were titrated. (n = 3).
(TIF)

S1 Table Genes specifically upregulated in hPIV1-infected cells at 2 dpi compared to hPIV3-infeced cells as analyzed by GO Biological Process 2018. (XLSX)

S2 Table Genes specifically upregulated in hPIV1-infected cells at 10 dpi compared to hPIV3-infeced cells as analyzed by GO Biological Process 2018. (XLSX)

S3 Table Genes specifically downregulated in hPIV1-infected cells at 2 dpi compared to hPIV3-infeced cells as analyzed by GO Biological Process 2018. (XLSX)

S4 Table Genes specifically downregulated in hPIV1-infected cells at 10 dpi compared to hPIV3-infeced cells as analyzed by GO Biological Process 2018. (XLSX)
