# Supplementary figures and images for: Human parainfluenza virus type 1 regulates cholesterol biosynthesis and establishes quiescent infection in human airway cells
Source: PLoS Pathog. 2021 Sep 16;17(9):e1009908. doi: 10.1371/journal.ppat.1009908 (PMC8445407; doi:10.1371/journal.ppat.1009908)

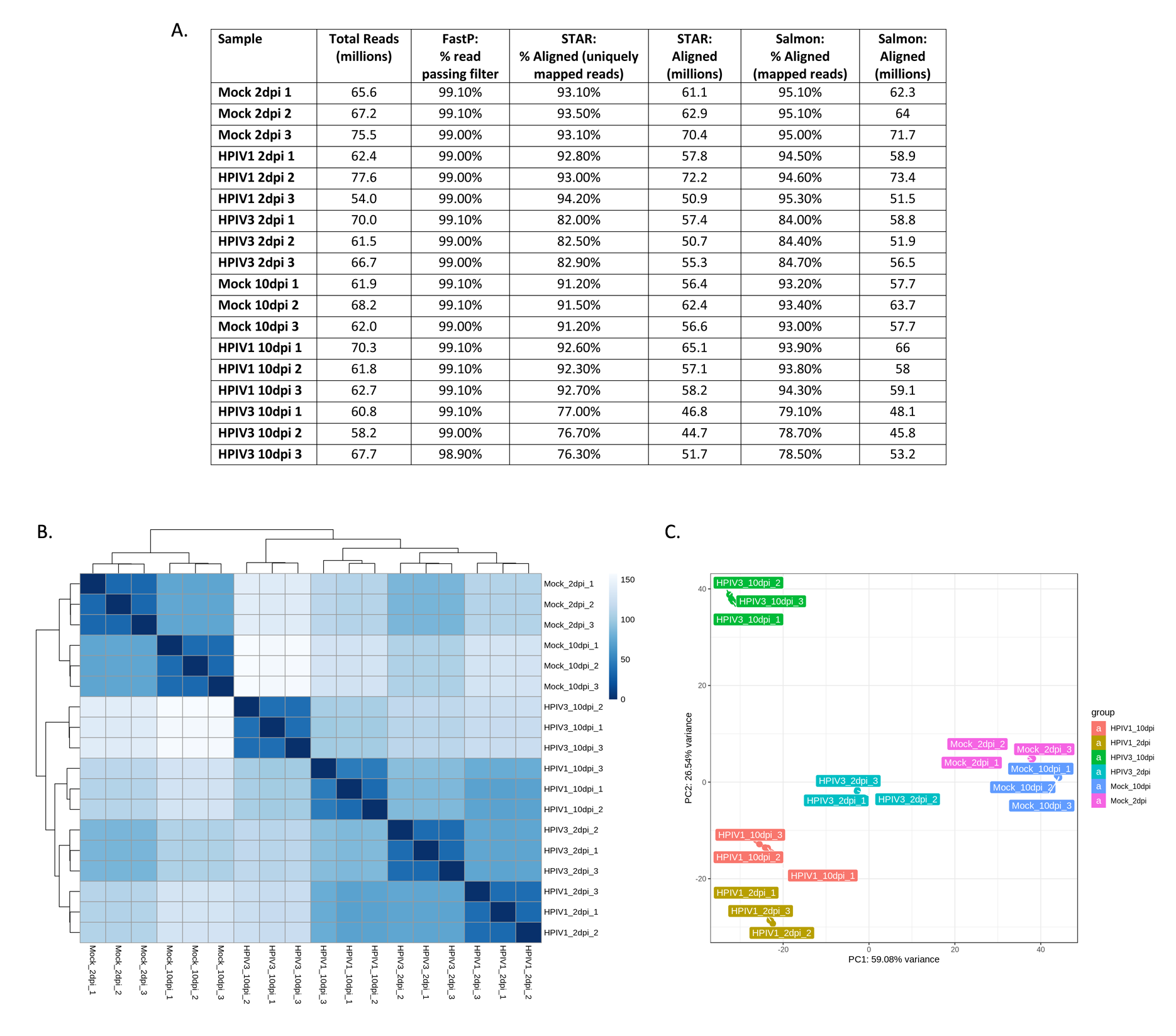

Supplement: S1 Fig — (A) Processing statistics for transcriptomic analysis of the 18 RNA samples including three biological replicates of uninfected (mock), hPIV1- or hPIV3-infected A549 cells cultured for 2 or 10 days. (B) Sample-to-sample distance matrix of RNA-Seq analysis. A heatmap shows the hierarchically clustered Euclidean distances between samples from the regularized log transformation of the normalized count data. The scale on the right demonstrates the arbitrary unit of distance between samples in which the dark color represents less distance (more similarity) and the light color represents greater distance between samples (less similarity). (C) Principal component analysis (PCA) plot representing the variance in the gene dataset. The samples shown in the 2D plane are spanned by their first two principal components of all samples including three replicates of mock, hPIV1-or hPIV3-infected conditions. (TIF) [file ppat.1009908.s001.tif]

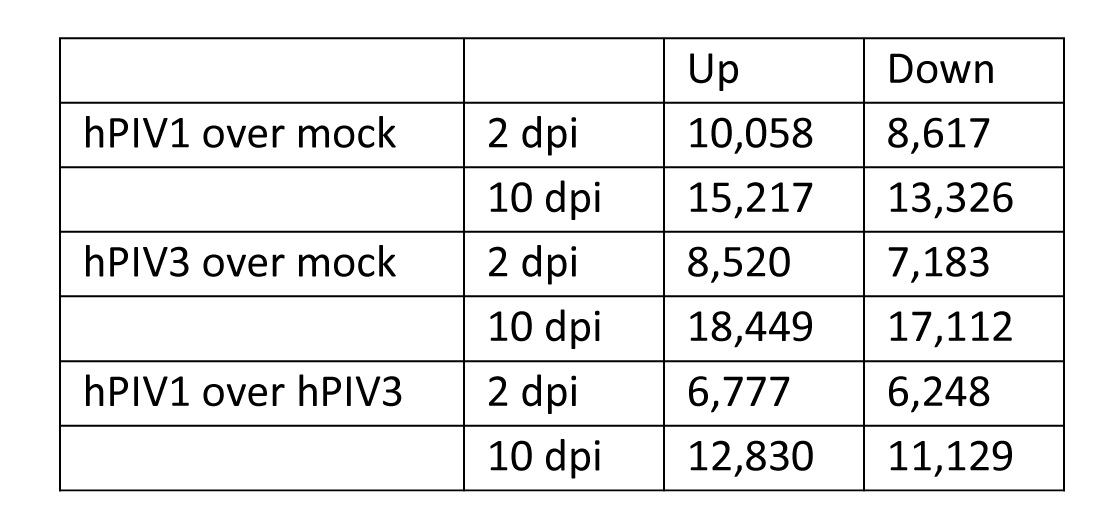

Supplement: S2 Fig — (TIF) [file ppat.1009908.s002.tif]

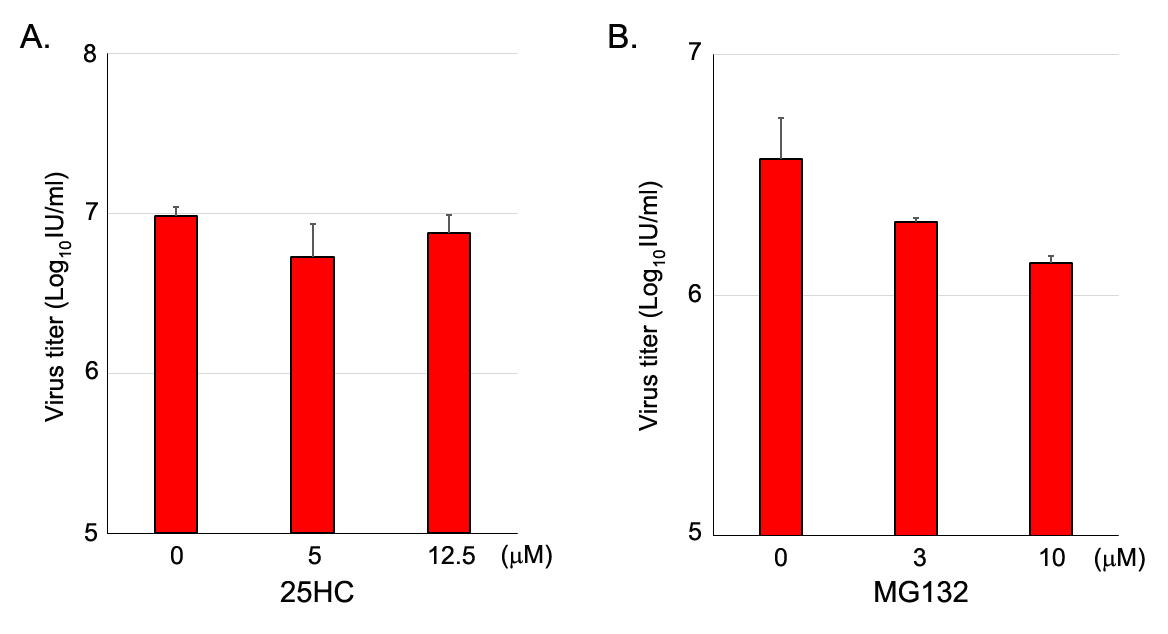

Supplement: S3 Fig — A549 cells were infected with hPIV1 at an MOI of 3 for 1 h and cultured in the presence of 25HC (A) or MG132 at indicated concentrations. Quantities of infectious virions in supernatants produced during 1–2 dpi (A) or 4–5 dpi (B) were titrated. (n = 3). (TIF) [file ppat.1009908.s003.tif]
